# Free-roaming dog populations and movement methodologies for global rabies elimination: knowns and unknowns – a scoping review

**DOI:** 10.3389/fvets.2025.1567807

**Published:** 2025-08-05

**Authors:** Laura Cunha Silva, Constanza Fellenberg, Jerónimo Freudenthal, Harish Kumar Tiwari, Salome Dürr

**Affiliations:** ^1^Vetsuisse Faculty, Veterinary Public Health Institute, University of Bern, Bern, Switzerland; ^2^Graduate School for Cellular and Biomedical Sciences, University of Bern, Bern, Switzerland; ^3^The University of Queensland, Brisbane, QLD, Australia; ^4^Faculdade de Medicina Veterinária, Universidade de Lisboa, Lisbon, Portugal; ^5^Indian Institute of Technology Guwahati, Guwahati, India; ^6^Sydney Medical School, Faculty of Medicine and Health, University of Sydney, Sydney, NSW, Australia; ^7^DBT Wellcome Trust India Alliance, Hyderabad, India

**Keywords:** rabies endemic countries, enumeration, zero by 30, disease elimination, dog-mediated rabies

## Abstract

Understanding free-roaming dog (FRD) demographics and movement patterns is essential for effective rabies control interventions, such as mass dog vaccinations (MDV). This review assesses published studies on FRD movement and enumeration to assess existing knowledge. A scoping review was conducted following PRISMA guidelines. Three databases, namely, Embase, Scopus, and Web of Science databases, were searched for publications between 2012 and 2024. A total of 2,167 articles were screened through successive filtration process to select a final corpus of 52 publications. The studies were predominantly from India (*n* = 8), Brazil (*n* = 6), Indonesia (*n* = 5), Guatemala (*n* = 5) and Chad (*n* = 5) and mostly investigated FRD population size. Several techniques were used for FRD enumeration, with photographic mark capture-recapture being the most common. Most FRD movement studies focused on home ranges, influenced by the technique and population size. In many studies, advantages and disadvantages of the techniques employed remained unreported, leaving a scope for misleading conclusions when comparing the methods used. The review highlights significant research gaps in FRD movement and population studies in rabies-endemic regions, which are often overlooked in rabies control strategies. Addressing these gaps through targeted research is essential for developing more effective, evidence-based interventions.

## 1 Introduction

Canine mediated rabies is a neglected disease, and its elimination is hampered by the lack of comprehensive data, particularly in resource-limited, rabies-endemic countries of Africa and Asia (1). Reliable epidemiological data are crucial to understand the disease burden, to implement and evaluate control measures, and to guide policy decisions (2). However, rabies-endemic countries often lack robust surveillance systems and face administrative barriers (2), resulting in obscuring the true impact of rabies leading to misallocation of resources and accentuating the neglect surrounding the disease (3). In addition to the limited availability of data, inadequate diagnostic capacity and a lack of political commitment and allocated financial resources make implementation of intervention measures such as mass dog vaccination (MDV) and human post-exposure prophylaxis (PEP) difficult (4, 5).

The dog populations that are generally uncontrolled in rabies endemic countries are referred to as free-roaming dogs (FRD) (1). Usually abundant around human settlements (6), FRD home ranges are reported to include sites such as schools, temples, shopping centers, community markets, and carcass disposal sites (7, 8). However, the movement patterns and population densities vary greatly within and between countries (9–12). Various factors, such as culture, beliefs, education, and urbanization, influence these characteristics of dog populations (13–15).

The assessment of existing knowledge of FRD abundance and their movement patterns can help to strategize rabies control interventions, such as vaccination coverage, and effectively manage their populations (9, 11, 16–21). Unfortunately, most interventions in endemic countries rarely consider the targeted dog population estimates (2, 22–24). For example, in India, animal birth control (ABC) programs rarely considered the FRD demographic composition (such as sex ratios and age structure), an oversight which results in little reduction in the FRD populations (25). Similarly, a study in Malawi and a population dynamics model have demonstrated that MDV campaigns against rabies frequently fail to achieve the recommended 70% coverage, partly due to a lack of understanding of the roaming behaviors and home ranges of FRD subpopulations (8, 26). These findings emphasize the need for more studies on FRD enumeration and movement patterns to inform critical preintervention strategies, such as defining target populations, identifying vaccination areas, and understanding FRD behaviors to enhance the effectiveness of rabies control efforts.

An array of techniques originally developed for assessing wildlife abundance are applied to estimate FRD population sizes and behavior. A systematic review from 2015 identified techniques used to estimate FRD abundance such as direct and indirect counts, capture-recapture methods, and radio telemetry studies (27). World Organization for Animal Health (WOAH) identifies two methods, direct observation and mark-resight, for determining FRD population size and lists the potential downfalls of each (28). Both techniques rely on assumptions of equal visibility of marked and unmarked dogs, and no change in FRD population in the survey area, which may not always apply (28). A 2013 published systematic review of methods for estimating the size of restricted domiciliary dog populations found these methods for FRD to be generally questionable due to measurement bias and biases associated with length of sampling time, selection bias and non-response bias (29).

In contrast to enumeration studies, there are no reviews or guidelines outlining methods for investigating FRD movement. However, this does not imply that methods for analyzing FRD movement are absent. Similar to enumeration studies, movement methods rely on techniques already widely used in ecology research (30–33). The increased accessibility of GPS techniques in recent years has led to a rise in movement studies and published literature on FRD, with many articles focusing on home range analysis (34–38) and contact network analysis (7, 39–42).

In many settings where funding for rabies control is limited, relying on existing data and proven tools, supported by adaptable, evidence-based guidelines, can be a more feasible and equitable path toward effective implementation. The objective of this scoping review is to provide a comprehensive overview of the various methods used to estimate FRD population sizes and movements. Despite the wide range of techniques available, there is still a lack of critical evaluation by the authors of the published articles regarding the methods they employed, including a clear understanding of the advantages and limitations of these techniques within their studies. This review seeks to address that gap by exploring the methodological approaches used in existing research and examining where the studies were conducted, reducing the need for each country to generate local evidence through resource-intensive research. Specifically, we analyze studies conducted between 2012 and 2024 to assess current knowledge in FRD enumeration and dog movement methodologies.

## 2 Materials and methods

This scoping review followed the PRISMA extension for scoping reviews (PRISMA-ScR) guidelines (43).

### 2.1 Information sources and search

A search for scholarly literature on the subject was performed through three electronic web-based literature databases: Embase, Scopus and Web of Science using the search string “[(Free-roaming OR Free-ranging OR stray) OR (Free AND (roaming OR ranging))] AND (dog* OR canine*) AND [behavior OR behavior OR movement OR (population AND (enumeration OR size OR estimat*))]”.

### 2.2 Eligibility criteria

To be included in the review, studies needed to focus on population estimation or movement of FRDs. Peer-reviewed journal articles were included if they were published from, and including, 2012 up to and including the end of 2024, written in English, French, Portuguese, or Spanish, and constituted original research involving observational studies on FRDs. Origins of the articles were systematically analyzed and only those from studies conducted in rabies endemic countries with sporadic dog-mediated human rabies, and dog-mediated human rabies as per the WHO – The Global Health Observatory (44) and Regional Plan for the elimination of canine rabies (45).

Studies were excluded if full text was not available, or if they concerned specific FRD groups (e.g., pregnant bitches, neutered dogs etc.). Reviews, commentaries, pre-prints, conference papers and opinion pieces were excluded from the review.

### 2.3 Selection of sources of evidence

**Figure 1:**
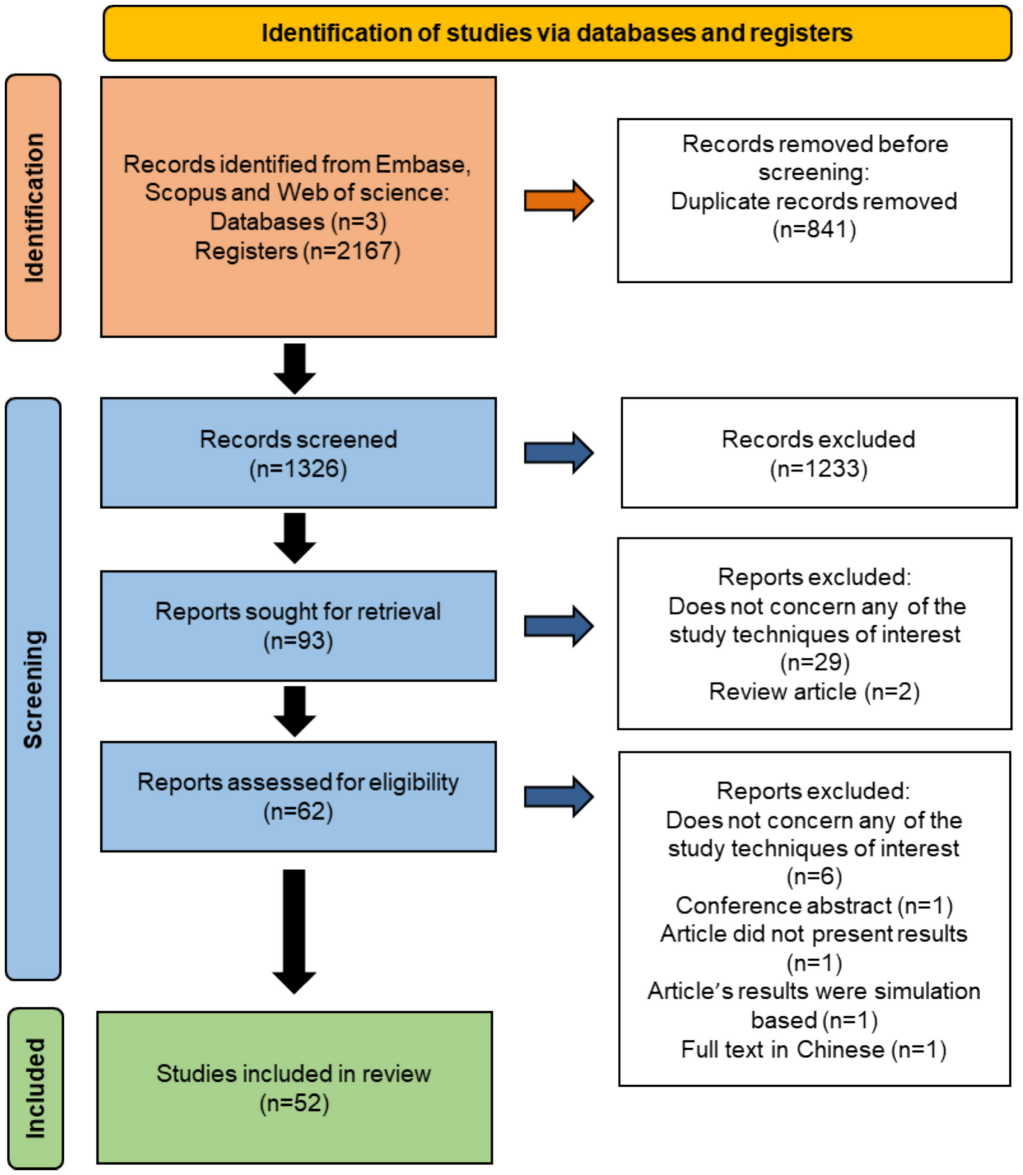
Prisma flowchart detailing the flow of information through the different phases of this scoping review. This chart showcases information on the number of records identified through literature search (Identification), number of records screened based on titles and abstract, and full text (Screening), and number of articles included in the scoping review (Included) and specific reasons for excluding articles after each selection process step.

A literature search following the criteria in section 2.1 and 2.2 identified 2,167 articles. After removing duplicates using Zotero,^1^ 1,326 articles remained (Figure 1). The articles were divided equally between authors LCS and CF, who independently screened titles and abstracts. Non-conforming articles were excluded based on inclusion and exclusion criteria, and disagreements were resolved through discussion or by consulting a third party (SD, HKT). Rayyan software^2^ was used for this process, resulting in 93 articles meeting the inclusion criteria.

Full-text screening was conducted by LCS, to exclude articles unrelated to dog population enumeration and movement techniques, thus narrowing the selection to 62 articles (Figure 1; Supplementary Table S1). During the data extraction step (section 2.4), and after consulting a third party (SD), 10 additional articles were again excluded (Supplementary Table S1), resulting in a final corpus of 52 articles.

### 2.4 Data charting and data items

A data-charting form was developed and discussed by the review team to identify key variables for extraction. Two researchers (LCS, JF) independently entered study characteristics, demographics, and other relevant data into an Excel spreadsheet, including details on study methodologies and the advantages and limitations of the methods as mentioned by the included articles’ authors. A list with data extracted is presented in Supplementary Figure S1. Preliminary extraction was tested with 20 items to ensure consistency. In cases of disagreement, a third researcher (SD or HKT) was consulted.

All data extraction was conducted using Microsoft Excel (Microsoft Corporation, Redmond, WA).

### 2.5 Synthesis of results

A descriptive analysis of the extracted data was conducted, including a narrative summary of key findings and article characteristics. Methodological differences, geographic variation, and sample sizes were considered when summarizing data. The advantages and limitations of methods used in enumeration and dog movement studies as stated by each included article authors were summarized in a table.

This study did not aim to conduct a meta-analysis; however, among movement studies, home range studies demonstrated consistent data collection processes, enabling comparisons of their applied methodologies. Median home range sizes presented in the articles were converted to hectares and illustrated with a bubble plot, showing variation by technique and study population size. Home range size variability across techniques was compared using the coefficient of variation (CoV) (46). Analyses were performed using R Statistical Software (v4.3.2; R Core Team 2023).

## 3 Results

### 3.1 Temporal and geographic distribution of the articles

**Figure 2:**
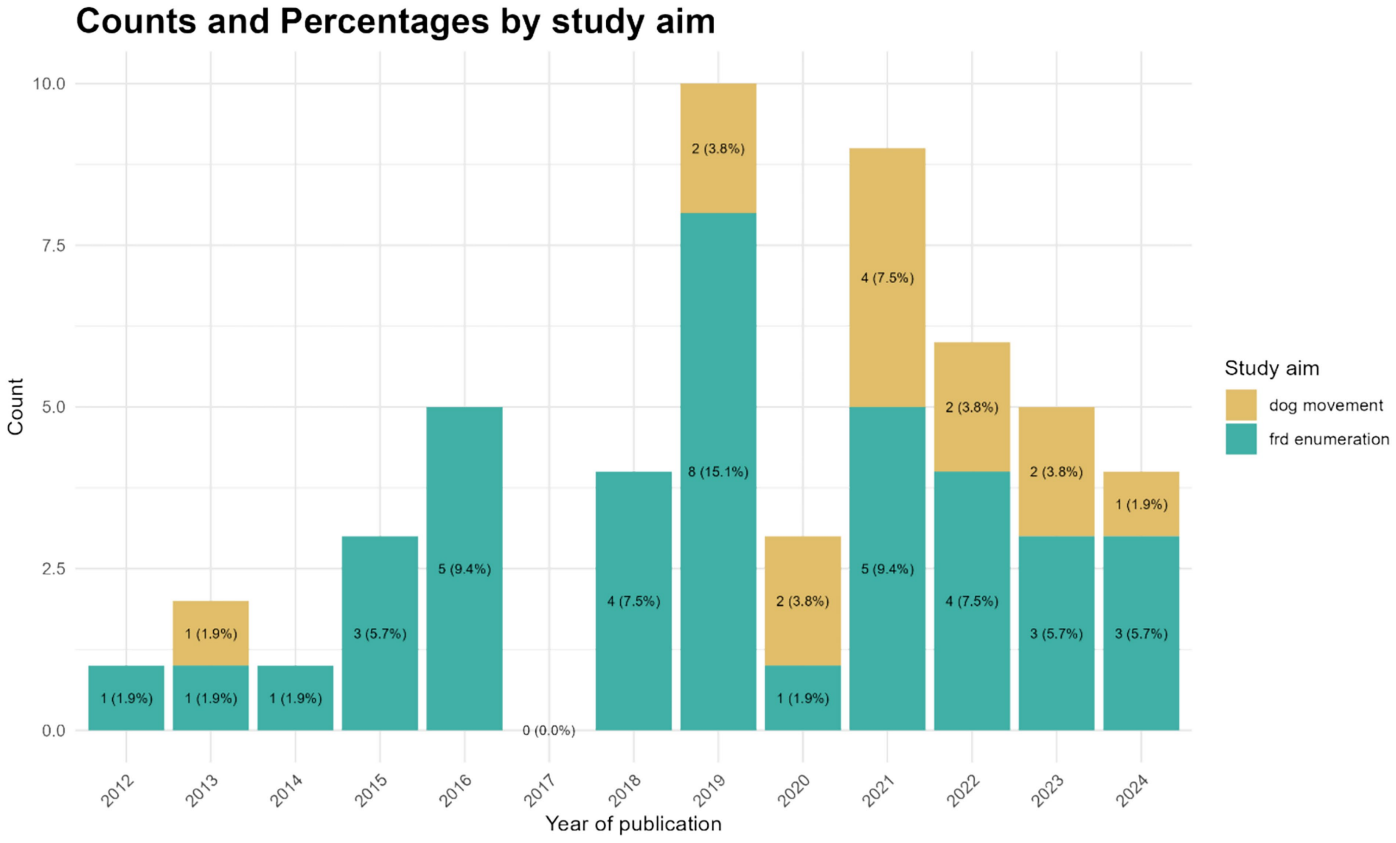
Barplot representing the number of articles per year and their primary focus of research. Articles were identified during a scoping review on dog population enumeration and movement in rabies endemic countries published between 2012 and 2024.

A total of 2,167 articles meeting our search criteria were identified, with 518 from Embase, 637 from Scopus, and 1,012 from Web of Science. Following the full screening process, 52 articles were ultimately included in this review (Figure 1). The majority of included articles were from 2019 (*n* = 10), closely followed by 2021 (*n* = 9), while the fewest articles were recorded in 2012, 2013 and 2014 (*n* = 1 each) (Figure 2). No article from 2017 was included. Most of the articles focused on FRD enumeration (*n* = 39), whereas 14 articles addressed FRD movement investigations. One study (47) utilized enumeration and movement techniques simultaneously (Figure 2). Articles specifically centered on dog movement were reported mostly after 2018.

**Figure 3:**
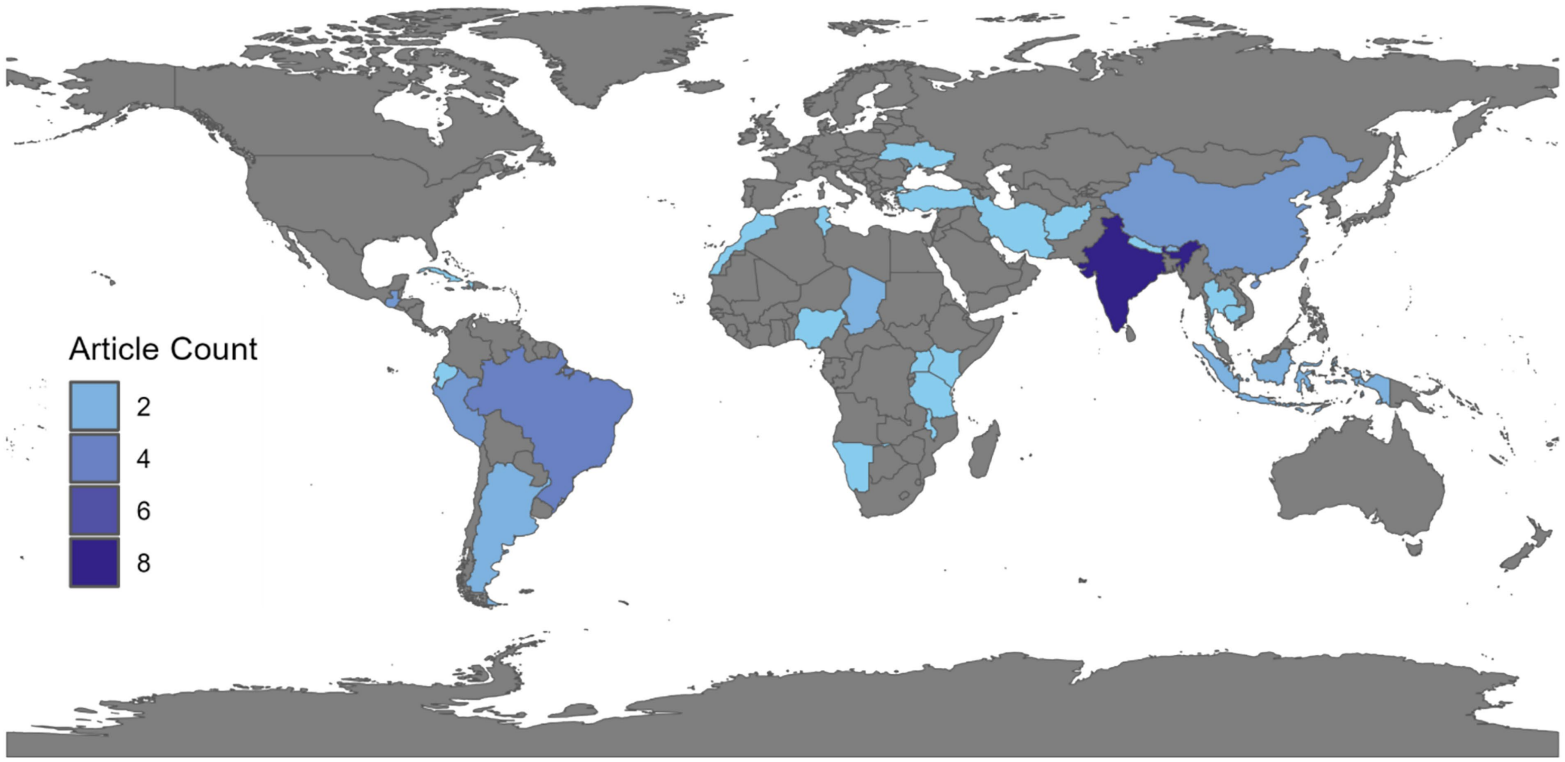
Choropleth Map depicting the number of articles published per country in the world. Articles were identified during a scoping review on dog population enumeration and movement in rabies endemic countries published between 2012 and 2024.

The 52 articles included in this analysis originate from 27 distinct countries. A significant portion of the articles were conducted in India (*n* = 8), Brazil (*n* = 6), Indonesia (*n* = 5), Guatemala (*n* = 5) and Chad (*n* = 5) (Figure 3). Notably, most countries (17 out of 27) reported only a single study conducted within their respective locations.

Twenty-two (56%) of the articles investigating FRD enumeration were conducted in urban sites, 13 (33%) in both rural and urban settings, and four (10%) exclusively in rural areas. Most (*n* = 6) movement articles occurred in both rural and urban areas (43%), whereas four movement articles (29%) were conducted in exclusively urban or rural areas.

Majority of included articles focused on both unowned and owned FRD (*n* = 35, 67%), whereas 14 (27%) focused specifically on owned FRD and three (6%) on unowned FRD.

### 3.2 Datasets and techniques used for FRD enumeration and movement studies

The datasets used for the FRD enumeration articles include transect and household surveys (*n* = 33), human population census (*n* = 6), dog census (*n* = 2), and photos from manual cameras (*n* = 16), unmanned aerial vehicles (such as drones, *n* = 1) and camera traps (*n* = 1). All articles on dog movements utilized GPS data, whereas direct observations and photographs were used in one study each on top of the GPS data.

All articles used between one and three different techniques within a single study (mean = 1.5 techniques). Photographic mark capture-recapture emerged as the most frequently used technique in FRD population estimates (*n* = 19) as well as dog:human ratio (*n* = 19), followed by simple transect count (*n* = 9) and mark-capture-recapture technique (*n* = 8). For FRD movement articles, the primary technique employed was the Minimum Convex Polygon (MCP) method (*n* = 8).

The datasets and techniques used in the included articles are presented in Table 1 for the enumeration studies and Table 2 for the movement studies. Supplementary Tables S2, S3 provide a detailed summary of each included article, covering its purpose, statistical methods, datasets, advantages, and limitations. Supplementary Table S4 offers a brief overview of the techniques used in the articles. Advantages and limitations to each technique have been reported by the authors of the included articles (Tables 1, 2). Most articles did not report any advantages (*n* = 12, 16%), limitations (*n* = 9, 12%), or both (*n* = 25, 32%) to their deployed techniques (Supplementary Tables S2, S3).

**Table 1:** Overview of the techniques for FRD population’s enumeration and their required dataset, advantages and limitations used in 39 articles identified during a scoping review between 2012 and 2024.

Dataset	Advantages	Limitations	References
Simple transect count, 9 articles			
Transect survey Household survey	Proved to be an effective and straightforward method for individually identifying the FRD within the selected area. Quick and relatively low-cost, while yielding indicative population estimates for FRD and generating valuable demographic data for dogs.	Can introduce biases as this method can only provide indicators of canine abundance rather than precise estimates of population parameters of abundance. Possible underestimation of the number of stray dogs due to confusion between owned, unchained or unconfined dogs, and actual stray dogs, particularly when there’s a significant proportion of owned but unchained or unconfined dogs in the area. Despite consistent methodology, variable detectability of free-roaming dogs (FRD) across sites may be a potential limitation. Counts are likely affected by socio-economic parameters and site topography. Possibility of double counts of the same dogs.	Tiwari et al. (55) Flores et al. (57) Tiwari et al. (54) Ochoa et al. (82) Peña et al. (56) Wu et al. (83) de la Reta et al. (84)* Carolina Chávez et al. (85)* Tavlian et al. (86)
Mark Capture-recapture, 8 articles			
Household survey Transect survey Post-vaccination transects School-based surveys Unmanned Aerial Vehicles (UAV) transect	Familiarity with the local dog population facilitates identification of dogs leading to more accurate statistics. Practical method for estimating the number and distribution of a free-roaming dog population if the assumption of a closed population holds true during the primary and secondary sampling intervals. This method is quick, cost-effective, and can be utilized for population surveys or to mark dogs during vaccination campaigns to assess vaccination coverage when other methods are unavailable or impractical.	Requires animal capture and manipulation. Assumes that the proportion of marked individuals resighted in subsequent samples represents the proportion of marked individuals in the entire population. When data is derived from CNVR (Catch, Neuter, Vaccination and Release) bias can be introduced since only voluntarily brought owned dogs to the clinic are counted. Reliability of the stray dog population estimate depends on the accuracy of owned dog estimates. Transects may result in same dog recounting and are less effective in larger sites. The method of marking dogs with paints may introduce bias (akin to trap-shy) and disturbs FRD natural behavior. Resource-intensive, limiting their use for regular population studies. Repeated direct counts along prescribed routes do not provide total abundance information. May not adequately estimate abundance due to differences in dog detection. Mark loss leads to misclassification. Difficulties with dark colored dogs and misclassification of colors may also occur. May not be suitable in countries where many dogs are kept indoors. Identification of owned, unchained dogs during street surveys based on WSPA protocols can be challenging. Motorized approach may compromise thoroughness of the search, contributing to underestimation of free-roaming dog counts by the sight-resight approach. Precision of the estimates may be affected by small sample sizes.	Rinzin et al. (87) Meunier et al. (58) Tenzin et al. (88) Sambo et al. (91) Warembourg et al. (92) Wu et al. (83) Tenzin et al. (89) Gill et al. (59)
Photographic mark capture-recapture, 19 articles			
Transect survey Camera traps Household-level census	Mark-Resight Logit Normal Method is suitable for free-roaming dogs (FRD) as marks are individually identifiable. Digital photographs improve identification of dogs enhancing accuracy, by validating reports from field surveyors and improving dog identification, thereby yielding more robust estimates. Accommodates constraints of time, money, and logistics, often requiring artificial marking. The use of natural marks reduces costs by avoiding the need for artificial marking and handling of dogs, which can pose risks to researchers and introduce biases. It may also be particularly useful for estimating abundance at smaller spatial scales or within larger cities using randomly selected spatial sub-units. The photographic capture-recapture method is efficient and suitable for estimating the street dog population, requiring minimal personnel. Provides a quick, cost-effective way to gather demographic data, offering a reliable, minimally biased population size estimates and detection probabilities, essential for planning mass vaccination programs. Photography-based sight-resight methods are advantageous over methods involving capture and handling of dogs due to simplicity, safety, lower costs, and reduced risks to dog health and welfare. Sufficient to provide an initial estimate of the ownerless dog population in urban and rural sites, given a limited available time for dog counting. Excludes any count variation due to behavioral attributes like “trap-happy” or “trap-shy.” Utilized across various animal taxa to estimate population size.	Surveyor’s fatigue may introduce bias Detectability influenced by weather variations- Field surveyors may make errors in unique dog identification during the single-round survey (SRS) method, but their recall of marks may be more accurate than a photo-based method. The use of natural marks presents on individuals to estimate abundance assumes that the marked population is representative of the unmarked population in terms of sightability and that dog sightability is not influenced by the presence or absence of natural marks. Assumes free-roaming dogs (FRDs) as a closed population and does not estimate recruitment and removal rates, which describe population changes. May have limitations in covering relatively large populations at larger spatial scales due to the need to identify a fair proportion of the population as ‘marked’ before sampling. Sight-resight effectiveness can be influenced by the landscape, and obtaining high-quality photographs may not always be feasible. Identifying and reidentifying dogs with less distinctive features can be difficult, leading to potential misidentification and reduced result accuracy. Sight-resight requires involving multiple people, facilities, supplies, and costs which pose a serious challenge for repeating the work. Usage for detecting changes in population size may require extended study periods to distinguish between population reduction and natural fluctuations. May be limited in populations with a high proportion of indistinct individuals. Requires at least two surveys. Concerns about the method’s applicability for long-term monitoring, as issues such as low light conditions affecting photo quality and individual identification accuracy need to be considered.	Tiwari et al. (55), Paschoal et al. (93) Cleaton et al. (63) Punjabi et al. (94) Shamsaddini et al. (60) Özen et al. (95) Silva et al. (96) Mustiana et al. (97) Smith et al. (62) Dias et al. (47) Tiwari et al. (54) Cárdenas et al. (61) Kalthoum et al. (98)* Bouaddi et al. (99)* Jagriti Bhalla et al. (100)* Emiliano and Adrián (101) Nasiry et al. (102) De Melo et al. (103) De Santi et al. (104)
Distance sampling technique, 2 articles			
Transect survey	May be suitable for enumerating dogs over large areas in a more time-efficient manner compared to the mark-resight approach. Does not require capturing or marking animals. May be a cost and resource-efficient method for estimating free-roaming dog populations since using only a representative number of roads for resight surveys may further save resources while maintaining an acceptable level of uncertainty in population abundance estimation. Could be valuable for resource-limited control programs, as it requires fewer resources. Method that can be easily applied by volunteers, enhancing method’s sustainability. Volunteers can reduce errors in data collection and provide sufficient information for management decisions. Direct observations of dog abundance (number of free-roaming dogs per kilometer) during street counts can serve as a reliable indicator of population changes and the effectiveness of management interventions.	The random placement of survey lines in distance sampling may not be valid when traveling along roads, potentially leading to an overestimation of dog abundance due to the association with roads and human activity. Assumes that all animals on the transect are detected and that detectability decreases with increasing distance. Hasn’t been widely applied for roaming dog populations and there is potential for mismeasurement of distances. Requires more computational expertise than capture-recapture methods for producing estimates.	Meunier et al. (58) Cárdenas et al. (61)
Dog:human ratio, 19 articles			
Household survey/census Transect survey Human census Dog census Estimated number of dogs from other technique Human: dog ratio currently used by the health authorities	Human-to-dog ratio method and dog census (owned FRD) identify the same dog populations, enabling direct comparison of results. High dog:human ratio is associated with an increased risk of rabies transmission.	Often underestimate the population size of free-roaming and ownerless dogs Socio-cultural factors and variations in human population density across different countries influence the outcomes. Human-to-dog ratio methods typically encompass all types of owned dogs, including puppies, and do not account for ownerless dogs or those in local shelters, leading to an overestimation of the ratio which can have significant financial implications for planning future dog vaccination campaigns.	Warembourg et al. (92) Gill et al. (59) Özen et al. (95) Mbilo et al. (22) Rinzin et al. (87)* de la Reta et al. (84)* Cárdenas et al. (61)* Tenzin et al. (88)* Wu et al. (83)* Silva et al. (96)* Shamsaddini et al. (60)* Tenzin et al. (89)* Kalthoum et al. (98)* Bouaddi et al. (99)* Kwaghe et al. (105)* Emiliano and Adrián (101) Nasiry et al. (102) Tenzin et al. (90) De Santi et al. (104)
Spatial models, 2 articles			
Transect survey GPS coordinates Geo-spatial data on study site	Spatial modeling integrating data from district level dog surveys offers a cost-effective and manpower-efficient alternative to nationwide dog surveys. The population distribution map generated from this approach can serve multiple purposes, including predicting dog numbers by incorporating factors like population structures and dynamics, forecasting disease occurrences like rabies within dog populations, and providing baseline data for dog population management plans. Spatial modeling serves as an alternative to address issues related to inappropriate sample sizes.	Results may not perfectly reflect reality. Interpreting and applying model predictions may requires local knowledge.	Thanapongtharm et al. (64) Tavlian et al. (86)

*No advantages or limitations presented.

**Table 2:** Overview of the techniques to investigate FRD movements and their purposes, required dataset, advantages and limitations used in 14 articles identified during a scoping review between 2012 and 2024.

Dataset	Advantages	Limitations	References
Purpose: Home range estimation			
Minimum convex polygon, 8 articles			
GPS Observation	Widespread use in estimating home range size and studying mammal ranging behavior, enabling comparison with existing literature. Yielded consistent HR patterns for free-roaming dogs. High accuracy in small sample sizes.	Prone to unpredictable biases that can impact results in comparative studies, particularly within species or populations. High sensitivity to extreme values. Prone to lead to overestimation of HR due to high dog activity at the time of the day when data was collected. Prone to generate reduced precision in HR calculation for very small number of observations per individual. Results are contingent upon the methodology of data collection, the specific home range calculation techniques employed, and the defined isopleth sizes.	Tiwari et al. (54) De la Puente-Arévalo et al. (8) Melo et al. (15) Cunha Silva et al. (6)* Wilson-Aggarwal et al. (107)* Dias et al. (47)* Ladd et al. (108)
Kernel techniques, 3 articles			
GPS	Auto-correlated kernel density estimates calculated from continuous time movement models (Ornstein-Uhlenbeck (OU) and Ornstein- Uhlenbeck with foraging (OUF)) enables a thorough examination of both fine- and broad-scale movement processes and avoids underestimation of space use.	Kernel density estimates often underestimate spatial usage.	Wilson-Aggarwal et al. (106) Wilson-Aggarwal et al. (107)* Dias et al. (47)*
Bayesian random bridge technique, 3 articles			
GPS	Widespread use in estimating home range size and studying mammal ranging behavior, enabling comparison with existing literature. Compatibility with irregular GPS data records due to parameter that corrects for this fact. Less affected by extreme values compared to MCP. More realistic predictions since it is based on animal movement tracts rather than location.	Need to define values for parameters (e.g., Hmin) that varies with GPS device accuracy, posing a challenge for result comparison across studies. Isopleth centroid points represent the arithmetic mean value for a two-dimensional distribution and are theoretical constructs, mainly affecting affects larger isopleths; Therefore, they do not provide insight into whether the animal was specifically attracted to a site or if its movement there was purposeful.	Warembourg et al. (21) De la Puente-Arévalo et al. (8) Muinde et al. (37)
Time-localized convex hull, 1 article			
GPS	Employment of “time-scaled distance (TSD),” i.e., nearest neighbors based on proximity in both space and time. Nearest neighbors determined by parameters inferred from the data. Well-suited for modern GPS data, which typically includes a time stamp along with the GPS coordinates.	No comment.	Raynor et al. (9)
Purpose: habitat selection			
Mixed effects logistic regression model, 1 article			
GPS	Can addresses spatial autocorrelation. Can consider the heterogeneous distribution of resources	No comment.	Cunha Silva et al. (6)
Purpose: contact networks			
Social network analysis, 2 articles			
GPS	Centrality metrics can be used as an indicator of disease transmission. Can be further used to inform agent-based disease transmission models that respect the heterogeneity between individual dogs.	Resource-intensive and impractical to undertake as default method to gain information on dog populations.	Warembourg et al. (21) Wilson-Aggarwal et al. (107)*

*No advantages or limitations presented. GPS = global positioning system.

### 3.3 Summary of home range sizes

**Figure 4:**
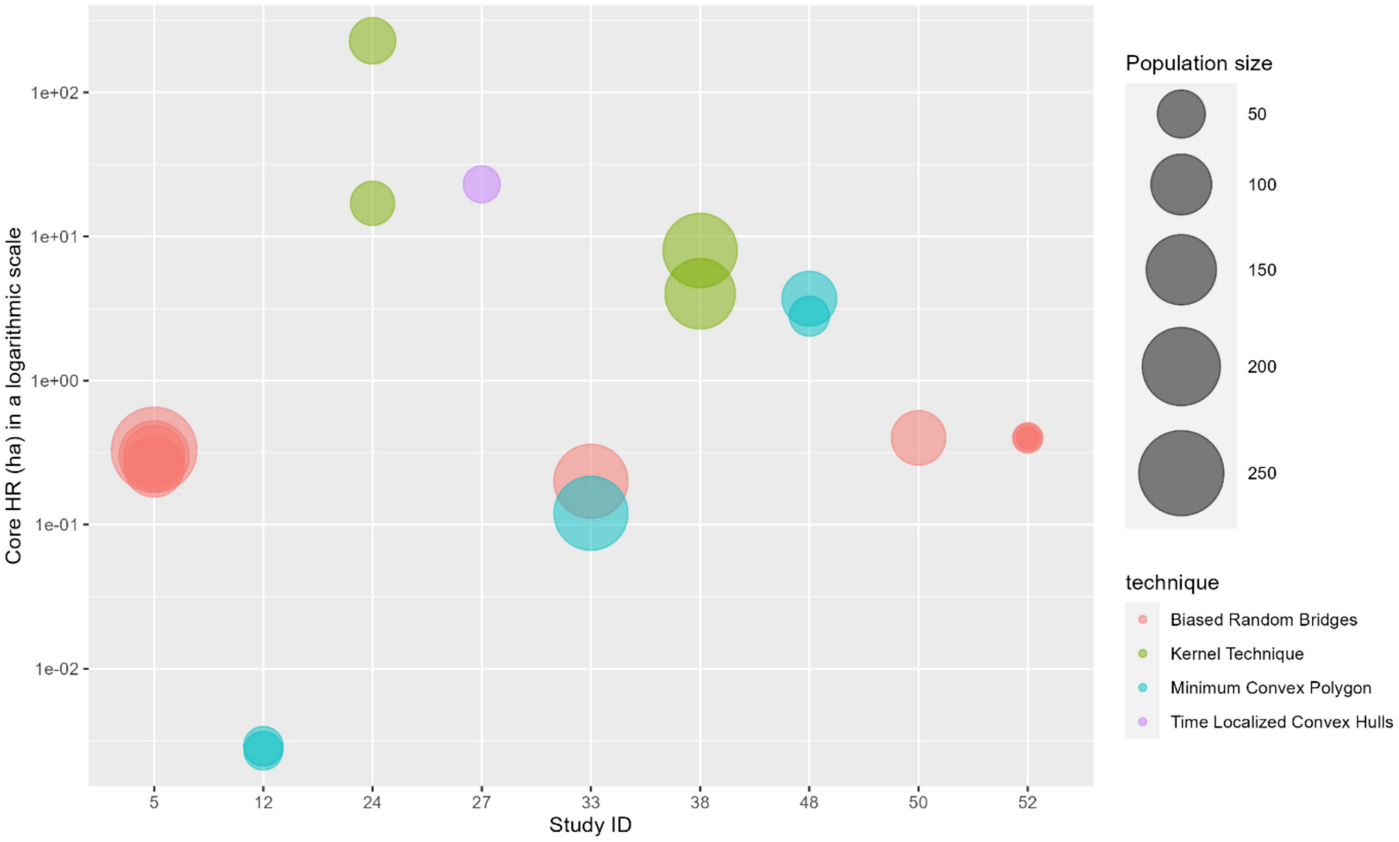
Bubble plot illustrating the estimated median core home range size (in hectares and in a logarithmic scale) in eight articles included in the scoping review, visually separated by the technique used. The bubble size represents the sample size of dogs included in the respective article.

**Figure 5:**
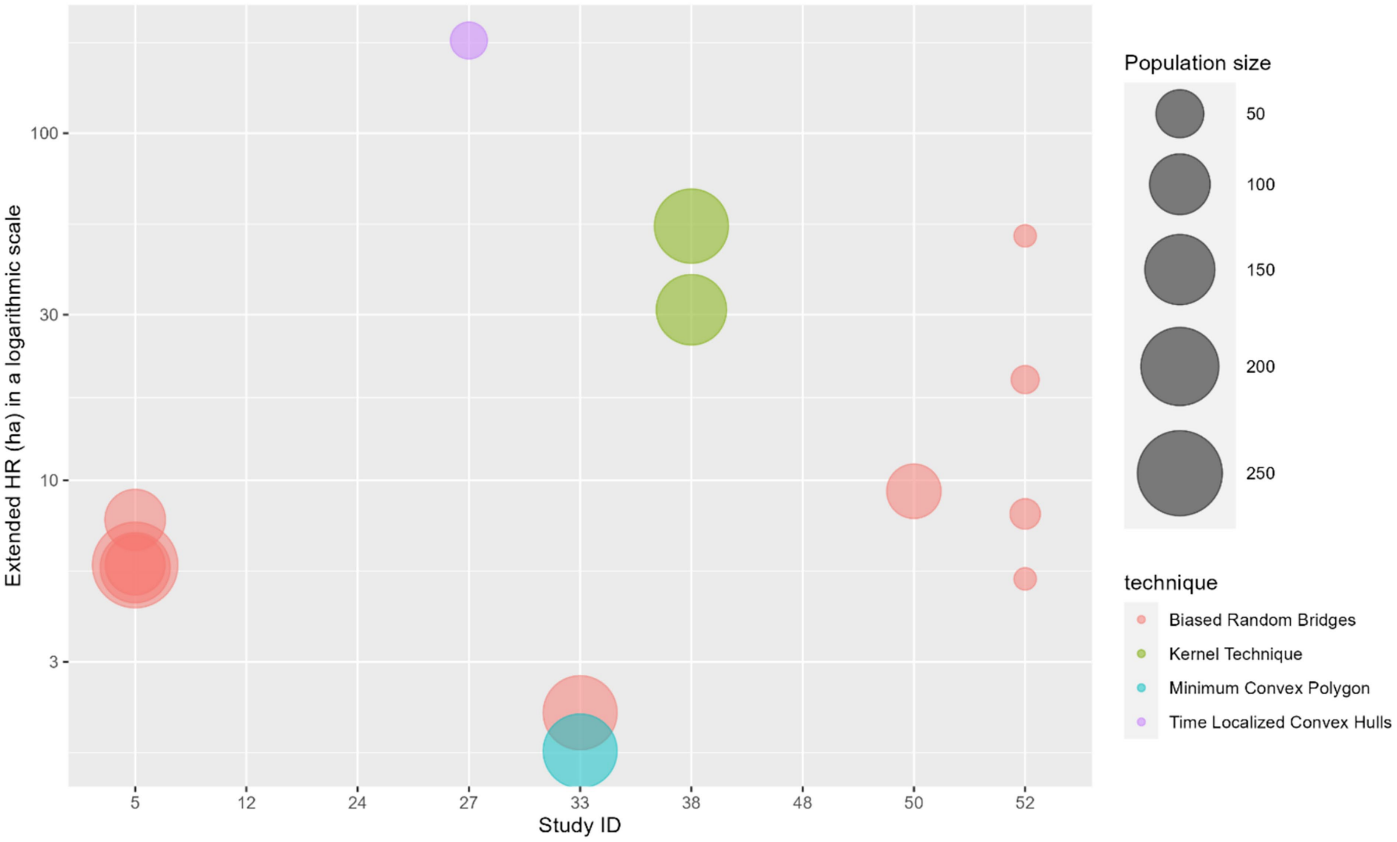
Bubble plot illustrating the estimated median extended home range size (in hectares and in a logarithmic scale) in eight articles included in the scoping review, visually separated by the technique used. The bubble size represents the sample size of dogs included in the respective article.

Home range estimation was conducted in 14 articles, with 11 providing analyzable results. These articles used minimum convex polygon (MCP) (*n* = 6), biased random bridges (BRB) (*n* = 5), kernel (*n* = 2), and time-localized convex hull (T-LoCoH) (*n* = 1) techniques. Five articles (Study ID 5,12, 38,48,52) applied the same method across different sites, while one article (Study ID 33) used both BRB and MCP at the same site. Median core home ranges ranged from 0.0027 to 228 ha (mean = 11.6 ha), and extended home ranges from 1.66 to 2,400 ha (mean = 258.6 ha). Variation is high, both for core and extended HR values. The BRB showed the most consistent core home range results (CoV = 0.22), while MCP had the high variability for both core (CoV = 1.35) and extended home range estimates (CoV = 1.01), and kernel showcased the highest variability for core home range estimates (CoV = 1.70) (Figures 4, 5).

## 4 Discussion

The first milestone of the rabies elimination roadmap, defined by organization United Against Rabies, is building evidence related to various fields concerning dog rabies elimination, including the abundance and behavior of FRD (48). Yet, it is evident from our review that rabies endemic countries in Africa and Asia largely lack compiled information in the scientific literature on dog populations. Among the 81 countries worldwide that are considered endemic for dog-mediated rabies, i.e., countries with present or sporadic dog-mediated human rabies (44, 45), we have identified only 27 (33%) that have conducted studies on either FRD population estimates or movement. Among these 27 countries, 17 (63%) have only one scientific article published within their borders. This underscores a notable gap in local knowledge and a lack of understanding of the diversity in FRD populations and movement patterns between and within countries. Most action plans in rabies endemic countries exclude the need for understanding FRD movement, behavior and demography for effective intervention. In addition, the approach to apply Oral Rabies Vaccination (ORV) campaigns to poorly accessible FRD, as discussed by the WHO (49), is promoted in guidelines, but often disregarded. This can both be an effect, or a reason for the scarcity of dog population studies conducted in rabies endemic countries.

Within the two continents mostly affected by rabies, our review included fewer studies from African countries compared to Asian countries. The included 11 Asian countries represent 44% of the 25 Asian countries endemic for dog-mediated human rabies, whereas in Africa only 21% (9 out of 42) of rabies endemic countries were represented in the review (44). This finding may stem from each continent’s policies regarding rabies and FRD or dogs in general, which are largely lacking or if present, are not effectively implemented (50–53). When compared with the number of studies identified from outside Africa and Asia, we found that studies originated from 11 (79%) out of the 14 rabies endemic countries. This indicates that these countries, located mainly in Latin America, invested more into FRD research, and thus rabies control (45). Within Asia, India and Indonesia collectively produce 13 out of the 26 total articles (Figure 3). The heterogeneous distribution observed may be attributable to several factors, including a higher actual or perceived burden of FRD and rabies in these regions, larger human population sizes, or the presence of active research groups in these countries. Indeed, a single research group is responsible for at least half of the included publications from India, while another research group contributed the majority of included articles from Indonesia.

Estimating FRD population size is challenging and prone to bias. This is mainly due to the heterogeneity in the dog population, i.e., the presence of both owned and ownerless dogs, leading to variability in detection probabilities (27, 54, 55). Therefore, trade-offs between complexity in study design and data analysis, and simplification with potentially higher risk of bias need to be considered. For example, the simplest technique deployed, the dog:human ratio calculations, was used 19 times. However, it was typically presented as secondary results rather than being the primary focus of an article on dog population estimates. Ratio estimations are influenced by variations in human population density, making it difficult to make such findings universally usable (27). Also, simple methods like transect counts and distance sampling are effective and cost-efficient. However, both techniques do not consider heterogeneous probabilities of animal detection (27, 56). More specifically, simple transect counts only provide indicators of canine abundance rather than precise population estimates (57), while distance sampling’s random line placement on long roads can lead to overestimations (58).

Most studies on FRD enumeration (27 out of the total) have employed capture-recapture methods. Despite their common use in research, capture-recapture techniques assume a closed population, which was reported as a limitation in the here assessed articles (13, 47, 58–62). A closed population is only met if studies are conducted over short periods with negligible immigration and emigration of dogs, no loss of marks, no misclassification between marked and unmarked dogs, and homogeneous capture probabilities (27). This may be realistic for some, but not all studies conducted. The marking of the dogs can be done individually (e.g., by photos taken), or overall (e.g., by marking them with collars or paint without differentiating between individuals). The main advantage of using photography for marking FRD is that it accounts for individual heterogeneity, providing more accurate estimates compared to simple mark capture-recapture (55). Additionally, digital photography reduces costs associated with artificial marking, avoids handling dogs, and eliminates count variations due to trap-shy or trap-eager behavioral response. However, photographic capture-recapture has limitations, as recognizing individual FRD can be challenging and potentially limited to populations with many indistinguishable individuals (54, 60, 62, 63). Investment into research on more resource-friendly approaches to match individual dogs in photographs is thus demanded. Simple mark-capture recapture is faster, and it does not require the laborious task of reviewing photographs and identifying individual dogs, making it less prone to human error and observer fatigue. Another technique reported are spatial models, which has been used in two studies (64). These complex models require preparing spatial data before analysis which is challenging and time-consuming, hence limiting its use, but holds promise due to their versatility and ability to handle small sample sizes.

Data collection for dog enumeration studies is diverse, drawing from multiple sources. Dog counts during transect surveys is one data collection method, which, despite their straightforward application, must account for factors like topography, climate conditions, and lighting, as these elements impact FRD detection, photographic capture-recapture, and distance measurements (Supplementary Tables S2, S3). Household or school-based surveys (including aiming for full censuses) are also common methods, but they are labor-intensive, often underestimating, and time-consuming (27).

Overall, it can be said that enumeration methods are becoming more diverse and complex, moving beyond simpler techniques. This complexity, coupled with a lack of recognition of the importance of FRD population studies in the development of National Action Plans (NAPs), may contribute to the limited number of studies on this topic (2). Additionally, the lack of a gold standard methodology for estimating free-roaming dog populations increases uncertainty and limits the comparability between study findings. In the absence of a gold standard, population estimation methods from wildlife have gained acceptance (54). Nonetheless, selecting the most suitable technique is challenging and largely influenced by the resources and conditions available at the study site, and by the limitations of the methods, which has been presented in this review (27).

Despite the importance of FRD movement patterns for rabies control and disease spread (9, 65), no consensus on a gold standard for FRD movement studies has been met. Also, so far, this is the first attempt to provide an overview on techniques used to investigate FRD movement behavior alongside the included authors’ stated advantages and limitations of deployed techniques. Most articles on this topic are published after 2019, and all included movement articles reviewed here rely on GPS-collected data. They emerged after a study published from Australia in FRD in Aboriginal communities, using GPS collars and comparing methods for home range calculations (34). The GPS technology allows researchers to study animal movements without human interference and in a non-intrusive manner (66, 67). Advances in this technology over recent years have made GPS units increasingly lightweight and small to carry (68). The decreasing cost and greater market availability of GPS technology, along with the enhanced computational power to process extensive GPS datasets, have made conducting movement studies more feasible (66). These advancements have minimized the need for labor-intensive observations by researchers, likely leading to an increase in such studies in recent years.

From the articles included in this review, we found that authors of movement studies primarily focus their efforts on estimating FRDs’ home ranges. We found that home range sizes are conditional to the type of technique and study population size used, with less complex techniques (such as the MCP) producing more inconsistent results (Figures 4, 5). The widespread adoption of simpler techniques in movement studies often overlooks either the spatial, temporal, or both complexities inherent in animal movement (69, 70), making it difficult to compare home range studies across different countries and regions. The BRB method was discussed to deliver the most reliable home range estimates, likely due to its highly effective method for addressing serial autocorrelation in movement data, frequent in animal tracking studies (71). Additionally, BRB’s ability to decompose spatial usage into frequency and repetition components allows gathering information on an animal’s number of visits to particular locations and the average time spent there (71). Such detailed spatial information is often lacking in simpler techniques like MCP and conventional kernel methods. This pattern is particularly evident in the results obtained using the MCP. Although MCP estimates exhibited substantial variability, they were among the lowest home range values reported, even across populations of varying sizes. This outcome is somewhat unexpected, given that MCP is known to be highly sensitive to outliers and typically tends to overestimate home range size due to the influence of a few wide-ranging individual fixes (69, 72). A plausible explanation for these findings is the lack of standardization in sampling protocols and inherent differences between dog populations (21). Regardless of population size, when sampling regimes (e.g., number of location fixes, tracking duration, spatial coverage) are not standardized, MCP estimates are prone to remain highly variable (72). In addition, values may vary according to the method specifications used to define core and extended home ranges, as these are determined by varying percentage thresholds applied to the sampled data (e.g., excluding the top 5% of outliers to calculate an extended home range encompassing 95% of all recorded fixes).

Research on habitat selection and contact networks of FRD is limited in number, revealing a significant knowledge gap in rabies-endemic countries. Despite some research on the impact of dog movement on rabies outbreaks from rabies endemic areas (8, 9, 37, 73), most such has been carried out in non-endemic regions like Australia (42, 74–78). Investing in habitat selection and social network analysis research of FRD in rabies endemic regions are thus needed to better guide rabies control interventions, such as where to deposit ORV and which dogs primarily to be targeted for vaccination in case resources are limited.

The number of articles identified that investigated FRDs’ movement in rabies endemic countries is limited. Additionally, many enumeration studies are often commissioned by government entities and remain unpublished, meaning that such information is not available in the scientific literature. There is, however, a growing trend among authors to consider the complexities of animal movement in time and space by selecting more sophisticated techniques and acknowledging the limitations of simpler techniques. In contrast, authors using these more advanced techniques rarely discuss their limitations.

We used a scoping review methodology rather than a systematic review due to the exploratory nature of the research question and the diversity of study designs and outcomes in the identified articles (79). We acknowledge that our search string and eligibility criteria may be restrictive, potentially excluding studies that investigated dog enumeration and movement but may have not been captured. However, the objective of this review was to provide a comprehensive overview of the methods currently employed in the scientific literature to study FRD movement and enumeration. By focusing on rabies endemic regions, this review enhances relevance of the research in this field and identified knowledge gaps in areas most affected by the disease. Furthermore, several included studies did not report the advantages and limitations of their techniques, restricting our ability to fully assess the authors’ understanding of the strengths and weaknesses of the methods they employed in their research.

We here presented a large range of studies on FRD populations and movement in rabies-endemic regions using diverse technologies. At the same time, it became pertinent that research is limited to selected countries, hindering the development of locally adapted rabies control strategies, as these require detailed understanding of the local dog populations (9, 80, 81). Moreover, the high resource demands of the techniques used, and the absence of standardized methods, complicates the design of future studies and the comparisons across studies. Nevertheless, it may not be essential for each country and region to conduct their own research on dog populations; instead, they can draw on existing studies for valuable insights. This approach can be enhanced by developing comprehensive guidelines that countries can adopt for their own context and implement effectively.

## Footnotes

^1^ https://www.zotero.org/

^2^ https://www.rayyan.ai/
