# Supplementary material for: Free-roaming dog populations and movement methodologies for global rabies elimination: knowns and unknowns – a scoping review
Source: Front Vet Sci. 2025 Aug 5;12:1567807. doi: 10.3389/fvets.2025.1567807 (PMC12363366; doi:10.3389/fvets.2025.1567807)
Supplement: Supplementary file 3 [file Table_2.docx]

Free-Roaming Dog Populations and Movement for Global Rabies Elimination: Knowns and Unknowns– A Scoping Review

Laura Cunha Silva^1,2^*, Constanza Fellenberg Canales^3^, Jerónimo Freudenthal^4^, Harish Kumar Tiwari^5,6,7§^, Salome Dürr^1§^

1 Veterinary Public Health Institute, Vetsuisse Faculty, University of Bern, Bern, Switzerland.

2 Graduate School for Cellular and Biomedical Sciences, University of Bern, Bern, Switzerland.

3 The University of Queensland, Brisbane, Australia.

4 Faculdade de Medicina Veterinária, Universidade de Lisboa, Lisboa, Portugal.

5 Indian Institute of Technology Guwahati, Guwahati, India.

6 Sydney Medical School, Faculty of Medicine and Health, University of Sydney, NSW, Australia.

7 DBT Wellcome Trust India Alliance Intermediate Fellow, Hyderabad, Telangana, India.

§contributed equally

Supplementary materials

Supplementary Table 1 - Reasoning for exclusion of articles upon further detailing of movement techniques to be included in this scoping review (Contact rates, Home range, Habitat selection) (from 84 articles to 53) and reasoning for exclusion of upon full-text screening (from 53 articles to 43 final included articles)

| Excluded paper title | Reason |
| --- | --- |
| Behavior of rural and urban free-ranging dogs in Viçosa, Minas Gerais, Brazil | Does not concern any of the relevant study techniques. |
| Abandonment of dogs in Latin America: Strategies and ideas | Does not concern any of the relevant study techniques. |
| Priorities for research into the impact of canine surgical sterilisation programmes for free-roaming dogs: An international priority setting partnership | Does not concern any of the relevant study techniques.  Review. |
| Dogs as Pets and Pests: Global Patterns of Canine Abundance, Activity, and Health | Does not concern any of the relevant topics |
| Large scale dog population demography, dog management and bite risk factors analysis: A crucial step towards rabies control in Cambodia | Does not concern any of the relevant techniques. |
| Baseline epidemiology and associated dog ecology study towards stepwise elimination of rabies in Kwara state, Nigeria | Does not concern any of the relevant techniques. |
| A cross-sectional study of health-related issues in relation to housing environment in a rural area of Amritsar, Punjab | Does not concern any of the relevant study techniques. |
| The Spread of Rabies Among Dogs in Pranburi District, Thailand: A Metapopulation Modeling Approach | Does not concern any of the relevant study techniques. |
| Circadian rhythm in behavioral activities and diurnal abundance of stray street dogs in the city of Sambalpur, Odisha, India | Does not concern any of the relevant study techniques. |
| A behavioral study of stray dogs in temples in Nakhon Ratchasima municipality, Thailand | Does not concern any of the relevant study techniques. |
| Factors of rabies maintenance in dog population in Kinshasa, Democratic Republic of Congo (DRC) | Does not concern any of the relevant study techniques. |
| Stray dogs in urban fragments: Relation between population's perception of their presence and socio-demographic factors | Does not concern any of the relevant study techniques. |
| Free-ranging dogs prefer petting over food in repeated interactions with unfamiliar humans | Does not concern any of the relevant study techniques. |
| Free-ranging dogs show age related plasticity in their ability to follow human pointing | Does not concern any of the relevant study techniques. |
| The demography of free-roaming dog populations and applications to disease and population control | Does not concern any of the relevant study techniques. |
| To be or not to be social: foraging associations of free-ranging dogs in an urban ecosystem | Does not concern any of the relevant study techniques. |
| Assessing the impact of free-roaming dog population management through systems modelling | Does not concern any of the relevant study techniques. |
| Adjustment in the point-following behaviour of free-ranging dogs – roles of social petting and informative-deceptive nature of cues | Does not concern any of the relevant study techniques. |
| Human-Dog Relationships across Communities Surrounding Ranomafana and Andasibe-Mantadia National Parks, Madagascar | Does not concern any of the relevant study techniques. |
| Free-Ranging Dogs Understand Human Intentions and Adjust Their Behavioral Responses Accordingly | Does not concern any of the relevant study techniques. |
| Canine Conundrum: domestic dogs as an invasive species and their impacts on wildlife in India | Does not concern any of the relevant study techniques. |
| Anthropogenic food subsidy to a commensal carnivore: The value and supply of human faeces in the diet of free-ranging dogs | Does not concern any of the relevant study techniques. |
| Community perception and attitudes about the behavior of stray dogs in a college campus | Does not concern any of the relevant study techniques. |
| Demography of domestic dog population and its implications for stray dog abundance: a case study of Omsk, Russia | Does not concern any of the relevant study techniques. |
| Factors influencing intergroup agonistic behaviour in free-ranging domestic dogs (Canis familiaris) | Does not concern any of the relevant study techniques. |
| A dog's day with humans-time activity budget of free-ranging dogs in India | Does not concern any of the relevant study techniques. |
| Sex and physiological state influence the rate of resource acquisition and monopolisation in urban free-ranging dogs, Canis familiaris | Does not concern any of the relevant study techniques. |
| Differential foraging strategies: Motivation, perception and implementation in urban free-ranging dogs, Canis familiaris | Does not concern any of the relevant study techniques. |
| Population estimation methods for free-ranging dogs: A systematic review | Systematic review |
| Population estimation methods for free-ranging dogs: A systematic review | Systematic review |
| The spatio-temporal impact of domestic dogs (Canis familiaris) on giant panda (Ailuropoda melanoleuca) in Baishuijiang National Nature Reserve | Does not concern any of the relevant study techniques. |
| Dog Ecology and Rabies Knowledge of Owners and Non-Owners in Sanur, A Sub-District of the Indonesian Island Province of Bali | The use of a study technique is not a main component of the study. |
| Estimating stray dog populations with the regression method versus Beck’s method: a comparison | Simulation |
| If they could choose: How would dogs spend their days? Activity patterns in four populations of domestic dogs | Does not concern any of the relevant study techniques. |
| Participatory methods for the assessment of the ownership status of free-roaming dogs in Bali, Indonesia, for disease control and animal welfare | Does not concern any of the relevant study techniques. |
| Rapid community dog assessment in rabies endemic area | Conference abstract. |
| Time‑activity budget of urban‑adapted free‑ranging dogs | Does not concern any of the relevant study techniques. |
| Temporal activity of rural free-ranging dogs: implications for the predator and prey species in the Brazilian Atlantic Forest | Does not concern any of the relevant study techniques. |
| Response to short‑lived human overcrowding by free‑ranging dogs | Does not concern any of the relevant study techniques. |
| Parámetros demográficos en la población de canes y gatos domésticos en asentamientos humanos del distrito de Ventanilla, Callao-Perú | No results listed. |
| The spatio-temporal impact of domestic dogs (Canis familiaris) on giant panda (Ailuropoda melanoleuca) in Baishuijiang National Nature Reserve | Abstract in english but full-text in Chinese. |

Table S2 – Comments made by authors regarding solely the deployed methodology. This table details the type of dataset needed for method implementation as well as the author’s detailing for each method’s advantages and limitations on FRD enumeration articles.

| Purpose | Statistical method | Dataset | Advantages | Limitations | Number of studies which used this method | References |
| --- | --- | --- | --- | --- | --- | --- |
| **ENUMERATION FOR POPULATION ESTIMATES** | Spatial models | Transect survey | Offers a cost-effective and manpower-efficient alternative to manual dog surveys.  The population distribution map generated from our approach can serve multiple purposes, including predicting dog numbers by incorporating factors like population structures and dynamics, forecasting disease occurrences like rabies within dog populations, and providing baseline data for dog population management plans.  Spatial modeling serves as an alternative to address issues related to inappropriate sample sizes. | Results may not perfectly reflect reality. | 2 | Thanapongtharm et al., 2021 |
|  |  | Transect survey, GPS coordinates, geo-spatial data on study site | Relevant spatial datasets are increasingly available but remain underused for dog population estimates. Leveraging such data could offer valuable alternatives for estimating FRD population size.  These models can help guide vaccination strategies, resource allocation, and cost planning. They may also improve logistics, help evaluate vaccine coverage, and strengthen stakeholder support, all crucial for achieving national dog-mediated human rabies elimination. | In the absence of high-resolution geo-spatial data on the study site, interpreting and applying model predictions may requires local knowledge. |  | Tavlian et al., 2024 |
|  | Dog:human ratio | Household survey, transect survey | No comment. | No comment. | 19 | Rinzin et al., 2016 |
|  |  | Transect survey | No comment. | No comment. |  | de la Reta M. et al., 2018 |
|  |  | National census, Transect survey, photos | No comment. | No comment. |  | Cárdenas et al., 2021 |
|  |  | Transect survey | No comment. | No comment. |  | Tenzin et al., 2015 |
|  |  | Total human census, human: dog ratio currently used by the health authorities in Guatemala, household survey (dog census) | Human-to-dog ratio method and the dog census (transect survey) identify the same dog populations, enabling direct comparison of results. | Human-to-dog ratio methods typically encompass all types of owned dogs, including puppies, and do not account for ownerless dogs.  Often underestimate the population size of free-roaming and ownerless dogs |  | Warembourg et al., 2020 |
|  |  | Household survey, transect survey | No comment. | No comment. |  | Wu et al., 2021 |
|  |  | Transect survey, human population | No comment. | No comment. |  | Tenzin et al., 2015b |
|  |  | Adult human population data, transect surveys |  | Socio-cultural factors and variations in human population density across different countries influence the outcomes. |  | Gill et al., 2022 |
|  |  | Dog census, human population | No comment. | No comment. |  | Silva et al., 2019 |
|  |  | Free roaming dog  Population estimate, human population | No comment. | No comment. |  | Shamsaddini et al., 2022 |
|  |  | Human population, estimated number of dogs | No comment. | These estimates do not account for either owned dog populations or dogs in local shelters |  | Özen et al., 2016 |
|  |  | Household-level census, transect survey, photos | No comment. | No comment. |  | Kalthoum et al., 2021 |
|  |  | Household surveys | No comment. | No comment. |  | Bouaddi et al., 2018 |
|  |  | Household survey, population census | No comment. | No comment. |  | Kwaghe et al., 2019 |
|  |  | Household survey | No comment. | Method employed to estimate the dog population size does not include unowned dogs. This oversight likely results in an overestimation of the ratio, which has significant financial implications for planning future dog vaccination campaigns |  | Mbilo et al., 2019 |
|  |  | Free roaming dog  Population estimate, human population | No comment. | No comment. |  | Emiliano and Adrián, 2023 |
|  |  | Free roaming dog  Population estimate | No comment. | Results can vary significantly across regions and countries, influenced by sociocultural factors, environmental conditions, and dog-control strategies. |  | Nasiry et al., 2023 |
|  |  | Household surveys, human population census | No comment. | No comment. |  | Tenzin et al., 2024 |
|  |  | Free roaming dog  Population estimate , Human population census | No comment. | Comparing dog-to-human ratios can be difficult given differences in social, economic, demographic, environmental, and cultural factors. |  | De Santi et al., 2024 |
|  | Mark Capture-recapture | Household survey, transect survey | No comment. | When data is derived from CNVR (Catch, Neuter, Vaccination and Release) bias can be introduced since only voluntarily brought owned dogs to the clinic were counted.  Reliability of the stray dog population estimate depends on the accuracy of owned dog estimates.  The method of marking dogs with paints may introduce bias, as only docile dogs may be marked, potentially leading to an underestimation of the population size, while also causing physical disturbance to the dogs | 8 | Rinzin et al., 2016 |
|  |  | Transect survey | Repeated direct counts along prescribed routes are feasible for longitudinal studies. | Resource-intensive, limiting their use for regular population studies.  Repeated direct counts along prescribed routes do not provide total abundance information.  Requires animal capture and manipulation.  May not adequately estimate abundance due to differences in dog detection.  Mark loss leads to misclassification.  Difficulties with darkly colored dogs and misclassification of colors may also occur.  Assumes closed population and no mark loss |  | Meunier et al., 2019 |
|  |  | Transect survey | Assumes that the proportion of marked individuals resighted in subsequent samples represents the proportion of marked individuals in the entire population.  Lincoln-Petersen index is easy to implement.  Chapman estimate, using a modified algorithm, is less sensitive to population size and remains relatively simple to calculate.  Nonlinear logit-normal mixed effects model allows for population size estimation from multiple resight events.  Chapman estimate is less biased than the Lincoln-Petersen index. | Lincoln-Petersen index sensitive to overestimating population size.  Chapman estimate and Lincoln-Petersen index can only be calculated from one resight event per population.  Nonlinear logit-normal mixed effects model assumes closed population, free mixing of marked individuals, and stable marking. |  | Tenzin et al., 2015 |
|  |  | Post-vaccination transects, household surveys, school-based surveys | Post-vaccination transects yield more precise estimates than household or school-based surveys, but reliability depends on equal counting opportunities for all dogs.  Post-vaccination transects, combined with vaccination data, can rapidly generate and refine dog population estimates in areas with ongoing vaccination campaigns.  Transects may result in dog recounting and are less effective in larger villages or those with more subvillages. | Household surveys are limited to assessing owned dogs.  To estimate dog population a complete dog census, visiting each household in a community, is the gold standard but requires substantial time and resources, particularly in countries where the majority of dogs are owned.  Transects may not be suitable in countries where many dogs are kept indoors.  Estimates from household and school-based surveys are often inconsistent. |  | Sambo et al., 2018 |
|  |  | Unmanned Aerial Vehicles (UAV) transect, Transect survey and household survey | UAVs can detect ownerless dogs, owned free-roaming dogs, and visible confined dogs.  Advantages of using UAVs include autonomous flight planning, reproducibility, georeferenced data acquisition, low disturbance to wildlife, access to difficult areas, and higher coverage speed and area compared to foot transects.  Foot-patrol surveys using CR models provide satisfactory population size estimates and insights into population dynamics of free-roaming owned dogs. | Unmanned Aerial Vehicles (UAV) transect surveys and foot-patrol surveys are unable to detect the entire dog population.  The number of dogs detected with UAV surveys is substantially smaller than with foot-patrol transects, and quality of UAV pictures prevents differentiation between marked and unmarked dogs, hindering the utilization of capture-recapture models for population estimation.  UAV method is affected by picture quality, weather conditions, flight altitude, and dogs not being visible due to cover or distance from the UAV.  High workload for picture analysis, inability to detect all free-roaming owned dogs, and difficulty in differentiating confined and free-roaming dogs. |  | Warembourg et al., 2020 |
|  |  | Transect survey | Familiarity with the local dog population facilitates easier identification of dogs leading to more accurate statistics.  The motorized approach used in sight-resight surveys saves time for staff. | Identification of owned, unchained dogs during street surveys based on WSPA protocols can be challenging.  Motorized approach may compromise thoroughness of the search, contributing to underestimation of free-roaming dog counts by the sight-resight approach. |  | Wu et al., 2021 |
|  |  | Transect survey | Practical method for estimating the number and distribution of a free-roaming dog population if the assumption of a closed population holds true during the primary and secondary sampling intervals. | This method is quick, cost-effective, and can be utilized for population surveys or to mark dogs during vaccination campaigns to assess vaccination coverage when other methods are unavailable or impractical. |  | Tenzin et al., 2015b |
|  |  | Transect surveys | Considered a practical method for accurately estimating the number and distribution of a stray dog.  SuperDuplicates method is useful for estimating dog counts, especially for two sample surveys conducted on consecutive days. | Precision of the estimates may be affected by small sample sizes.  Positive (food baits) and negative (marking) reinforcements can influence dog behavior and sighting probabilities. |  | Gill et al., 2022 |
|  | Photographic mark capture-recapture | Transect surveys, photos | No comment. | Biases could arise when estimating total population size through extrapolation | 19 | Hu et al., 2019 |
|  |  | Transect survey, photos | Mark-Resight Logit Normal Method is suitable for free-roaming dogs (FRD) as marks are individually identifiable.  Methods considering individual heterogeneity yield more robust estimates. | Survey fatigue may decrease counts with increased surveys.  Planning enumeration surveys must consider forecasted weather and human events, as observer attentiveness affects detectability. |  | Tiwari et al., 2018 |
|  |  | Camera traps | Camera traps, a commonly used wildlife sampling method, can be used in estimating the population size of free-ranging dogs. | No comment. |  | Paschoal et al., 2016 |
|  |  | Transect survey, photos | Capture-recapture methods are particularly well-suited for estimating population sizes of free-roaming animals.  Improved identification of dogs, such as through digital photographs or better marking methods, can enhance accuracy, by validate reports from field surveyors and improve dog identification, thereby refining population estimates. | Capture procedures and marking techniques may scare off or traumatize animals, leading them to flee or hide from surveyors.  Capture-recapture methods require capturing and marking all visible animals in a defined area on day one.  Field surveyors may make errors in unique dog identification during the single-round survey (SRS) method, but their recall of marks may be more accurate than a photo-based method.  A photo-based method is time-intensive. |  | Cleaton et al., 2019 |
|  |  | Transect survey, photos | The mark-resight framework can be utilized in various circumstances, accommodating constraints of time, money, and logistics, often requiring artificial marking.  The use of natural marks reduces costs by avoiding the need for artificial marking and handling of dogs, which can pose risks to researchers and introduce biases. It may also be particularly useful for estimating abundance at smaller spatial scales or within larger cities using randomly selected spatial sub-units. | The use of natural marks present on individuals to estimate abundance assumes that the marked population is representative of the unmarked population in terms of sightability and dog sightability is not influenced by the presence or absence of natural marks.  Method may have limitations in covering relatively large populations occurring at larger spatial scales due to the need to identify a fair proportion of the population as 'marked' before sampling. |  | Punjabi et al., 2012 |
|  |  | Transect survey, photos | Sight-resight methods offer a quick and cost-effective way to gather demographic data for free-roaming dogs (FRD).  Photography-based sight-resight methods are advantageous over methods involving capture and handling of dogs due to simplicity, safety, lower costs, and reduced risks to dog health and welfare. | Sight-resight effectiveness can be influenced by the landscape, and obtaining high-quality photographs may not always be feasible.  Identifying and reidentifying dogs with less distinctive features can be difficult, leading to potential misidentification and reduced result accuracy.  Assumes FRDs as a closed population.  Sight-resight requires involving multiple people, facilities, supplies, and costs which pose a serious challenge for repeating the work. |  | Shamsaddini et al., 2022 |
|  |  | Transect survey, photo | No comment. | Challenges arise when previously marked animals cannot be identified due to marking degradation or when a subset of released animals cannot be recaptured. |  | Özen et al., 2016 |
|  |  | Transect survey, photos | The photographic capture-recapture method is suitable for estimating the street dog population.  This methodology has been demonstrated as efficient, requiring minimal personnel for execution, and can serve as an additional resource for planning actions to prevent and control urban zoonoses. | No comment. |  | Silva et al., 2019 |
|  |  | Household-level census, transect survey, photos | No comment. | No comment. |  | Kalthoum et al., 2021 |
|  |  | Household survey, transect survey, photos | Individual differences among dogs allow for recognition of individuals and determination of recaptures based on photographic evidence.  Sufficient to provide an initial estimate of the ownerless dog population in urban and rural sites, given a limited available time for dog counting. | Main limitation of dog counting is the utilization of a simple estimation method, which may not provide the most accurate results for estimating the unowned population. |  | Mustiana et al., 2015 |
|  |  | Transect survey, photos | Mark-recapture methods are commonly used for estimating population size, with closed methods allowing estimation of population size and detection probability, result in less biased parameter estimates.  Photographic methods for identification can reduce the impact of marking on detection probability and are advantageous over methods requiring physical contact. | Closed methods do not estimate recruitment and removal rates, which describe population changes.  Detecting changes in population size may require extended study periods to distinguish between population reduction and natural fluctuations.  Detecting changes in population size may require extended study periods to distinguish between population reduction and natural fluctuations.  The applicability of photographic mark-recapture methods may be limited in populations with a high proportion of indistinct individuals. |  | Smith et al., 2022 |
|  |  | Transects survey, photos | The two-sample method is suitable only for closed populations and involves one session of photographic marking followed by a single resight session. | The assumption of a closed population is necessary for the two-sample method to be valid. |  | Dias et al., 2013 |
|  |  | Transect surveys | No comment. | No comment. |  | Bouaddi et al., 2018 |
|  |  | Transect survey | No comment. | No comment. |  | Bhalla et al., 2021 |
|  |  | Transect survey, photos | Individual dogs were photographed from a distance without disturbing their natural behavior, thus excluding any count variation due to behavioral attributes like "trap-happy" or "trap-shy."  Can reliably produce a minimum estimate of the free-roaming dog population for planning mass vaccination programs. | Conducting surveys for 5–6 occasions is challenging, resource-intensive, and may introduce bias from surveyor fatigue. |  | Tiwari et al., 2019 |
|  |  | Transect survey, photos | The Capture-Recapture method (CR) is utilized across various animal taxa to estimate population size. | Requires two surveys.  Limitations of the CR method include the challenge of a closed population assumption and difficulties in identifying/marking individuals.  Concerns about the method's applicability for long-term monitoring, with issues such as low light conditions affecting photo quality and individual identification accuracy. |  | Cárdenas et al., 2021 |
|  |  | Transect survey, photos | Easy and inexpensive way to identify individual dogs over time without having to physically capture and mark them. | Assumes closed population, equal catchability among individuals, and mark retention throughout the study.  Possible misidentification of individuals through the photographs. |  | Emiliano and Adrián, 2023 |
|  |  | Transect survey, photos | Widely used methodology.  Cost-effective technique that reduces risks to researchers and minimizes disturbance to free-roaming dogs.  Digital photography aids in accurate individual identification.  Requires only two sighting sessions. | No comment. |  | Nasiry et al., 2023 |
|  |  | Transect survey, photos | It is a safe, fast, cost-effective option that eliminates the need for physical animal capture.  Allows for individual identification based on natural markings, enabling researchers to track recapture history.  When combined with GPS data to record locations, this method offers expanded analysis and a deeper understanding of free-roaming dog ecology. | Involves uncertainty in detecting all individuals and variations in encounter probabilities.  Accurate individual identification across all efforts is crucial for valid results.  Low photo quality or lack of detail can hinder accurate dog identification. |  | De Melo et al., 2023 |
|  |  | Transect survey, photos | No comment. | Assumes closed population, equal catchability among individuals, and mark retention throughout the study.  Some free-roaming dogs might have been misclassified as strays. |  | De Santi et al., 2024 |
|  | Simple transect count | Transect survey | Proved to be an effective and straightforward method for individually identifying the FRD within the selected area. | No comment. | 9 | Tiwari et al., 2018 |
|  |  | Transect survey | Method was chosen due to its speed and lower cost compared to other methods. | Can introduce biases.  This method can only provide indicators of canine abundance rather than precise estimates of population parameters.  Limitations of this method, such as underestimation of dog numbers, have been noted. |  | Flores et al., 2022 |
|  |  | Transect survey | No comment. | No comment. |  | de la Reta M. et al., 2018 |
|  |  | Transect survey | Quick and relatively low-cost, while yielding robust population estimates for FRD and generating valuable demographic data for dogs. | Despite consistent methodology, inherent difference in the detectability of free-roaming dogs (FRD) across sites may be a potential limitation. |  | Tiwari et al., 2019 |
|  |  | Transect survey | No comment. | Counts affected by socioeconomic strata in selected areas and site topography. |  | Ochoa et al., 2014 |
|  |  | Transect survey | No comment. | It is possible that many of the observations correspond to the same dogs. |  | Peña et al., 2016 |
|  |  | Transect survey | No comment. | No comment. |  | Chávez et al., 2016 |
|  |  | Household survey, transect survey | Questionnaire-based survey improve time and labor efficiency, especially in sites with relatively small dog populations and where local staff are familiar with the dog population. | Possible underestimation of the number of stray dogs due to confusion between owned, unchained or unconfined dogs, and actual stray dogs, particularly when there's a significant proportion of owned but unchained or unconfined dogs in the area. |  | Wu et al., 2021 |
|  |  | Transect survey | No comment. | Often underestimates the actual size of the FRD in a given area.  Can introduce selection bias. |  | Tavlian et al., 2024 |
|  | Distance sampling technique | Transect surveys | Distance sampling methods may be suitable for enumerating dogs over large areas in a more time-efficient manner compared to the mark-resight approach.  Does not require capturing or marking animals.  May be a cost and resource-efficient method for estimating free-roaming dog populations.  Despite requiring more computational expertise, distance sampling could be valuable for resource-limited control programs if shown to provide comparable results in other regions over large areas.  Using only a representative number of roads for resight surveys may further save resources while maintaining an acceptable level of uncertainty in population abundance estimation. | The random placement of survey lines in distance sampling may not be valid when traveling along roads, potentially leading to an overestimation of dog abundance due to the association with roads and human activity. | 2 | Meunier et al., 2019 |
|  |  | Transect survey, photos | Method that can be easily applied by volunteers, which is crucial to reduce errors in data collection and provide sufficient information for management decisions.  Direct observations of dog abundance (number of free-roaming dogs per kilometer) during street counts can serve as a reliable indicator of population changes and the effectiveness of management interventions. Moreover, this method requires fewer resources compared to others. | Assumes that all animals on the transect are detected and that detectability decreases with increasing distance. However, a limitation of this method, which hasn't been widely applied for roaming dog populations, is the potential mismeasurement of distances. |  | Cárdenas et al., 2021 |

Table S3 – Comments made by authors regarding solely the deployed methodology. This table details the type of dataset needed for method implementation as well as the author’s detailing for each method’s advantages and limitations on FRD movement articles.

| Purpose | Statistical method | Dataset | Advantages | Limitations | Number of studies which used this method | References |
| --- | --- | --- | --- | --- | --- | --- |
| **HR ESTIMATION** | MCP | Photos & GPS | Deploying GPS collars would offer more frequent fixes, improving home range assessment | Deploying GPS collars would result in a higher cost and potentially smaller sample size due to such costs | 8 | Tiwari et al., 2019 |
|  |  | GPS | MCP chosen due to its widespread use in estimating home range size and studying mammal ranging behavior, enabling comparison with existing literature | Potential bias in the MCP method can impact results in comparative studies, particularly within species or populations.  Significant differences in HR50 and HR95 estimates between MCP and BRB method.  MCP has a greater sensitivity to extreme values compared to BRB |  | De la Puente-Arévalo et al., 2021 |
|  |  | Observation & GPS | Yielded consistent HR patterns for free-roaming dogs.  MCP method is known for accuracy in small sample sizes therefore it provided precise enough HR values given the study's objective (evaluation of sterilization effects). | HR estimation via MCP method may lead to overestimation.  Study faced limitations in HR estimation since only a small number of FRD were captured/recaptured three times or more. The small available sample size reduces precision and statistical power. |  | Melo et al., 2020 |
|  |  | GPS | No comment. | No comment. |  | Cunha Silva et al., 2022 |
|  |  | GPS | No comment. | No comment. |  | Wilson-Aggarwal et al., 2019 |
|  |  | GPS | No comment. | No comment. |  | Dias et al., 2013 |
|  |  | GPS | MCP is a frequently used method for estimating an animal's home range, representing its entire activity area. | No comment. |  | Zhang et al., 2024 |
|  |  | GPS | No comment. | Variations in data collection, home range calculation methods, and isopleth sizes complicate direct comparisons of activity and home ranges via MCP. |  | Ladd et al., 2024 |
|  | BRB | GPS | Method previously recognized as suitable for FRDD GPS data. | No comment. | 3 | Warembourg et al., 2021 |
|  |  | GPS | Chosen due to widespread use in estimating home range size and studying mammal ranging behavior, enabling comparison with existing literature.  BRB was the primary choice due to its realistic approach and compatibility with GPS data recorded irregularly.  BRB method is less affected by extreme values compared to MCP, highlighting the importance of HR estimator selection in understanding free-roaming dog roaming behavior | Dependent on GPS fix frequency and device accuracy  HR sizes are influenced by Hmin value, which varies with GPS device accuracy, posing a challenge for result comparison across studies. |  | De la Puente-Arévalo et al., 2021 |
|  |  | GPS | Results in a more realistic animal movement pattern representation. | It is acknowledged that isopleth centroid points, representing the arithmetic mean value for a two-dimensional distribution, are theoretical concepts and do not correspond to real points.  Isopleth centroid points may not always be representative, particularly for polygons with wider areas within the 90% isopleths.  Isopleth centroid points do not facilitate inferences on whether the animal was directly drawn to a specific site or whether its movement there was purpose-driven |  | Muinde et al., 2021 |
|  | T-LoCoH | GPS | T-LoCoH surpasses classic LoCoH by employing "time-scaled distance (TSD)" instead of geographical distance to delineate local hulls around GPS points.  This approach is well-suited for modern GPS data, which typically includes a time stamp along with the GPS coordinates.  The advantage of T-LoCoH over LoCoH and previous methods lies in selecting nearest neighbors based on proximity in both space and time, with the weighting toward time proximity determined by parameters inferred from the data. | No comment. | 1 | Raynor et al.,2020 |
|  | Kernel techniques | GPS | Continuous time movement models were utilized for estimating home ranges due to evidence suggesting that conventional measures such as minimum convex polygons and kernel density estimates tend to underestimate space use.  Although only recently accessible to non-mathematicians, continuous time movement models to estimate space use enables a thorough examination of both fine- and broad-scale movement processes. | No comment. | 3 | Wilson-Aggarwal et al., 2021 |
|  |  | GPS | No comment. | No comment. |  | Wilson-Aggarwal et al., 2019 |
|  |  | GPS | No comment. | No comment. |  | Dias et al., 2013 |
| **HABITAT SELECTION** | Mixed effects logistic regression model | GPS | Addresses spatial autocorrelation.  Considers the heterogeneous distribution of resources | No comment. | 1 | Cunha Silva et al., 2022 |
| **CONTACT NETWORKS** | SNA | GPS | The centrality of a dog within a contact network can serve as an indicator of its potential contribution to disease transmission.  By integrating network analysis and disease modeling, it is feasible to pinpoint dogs with elevated risk of disease transmission based on their centrality metric. | Evaluating the structure of a contact network within a dog population is resource-intensive and impractical to undertake for every population before vaccination campaigns. | 2 | Warembourg et al., 2021 |
|  |  | GPS | No comment. | No comment. |  | Wilson-Aggarwal et al., 2019 |

Table S4 – Brief description of the techniques deployed in the included articles.

| **FRD enumeration techniques** |
| --- |
| - Simple transect count: Observers move along a line transects of known lengths and count all animals they see, without measuring distances (1). The results are expressed as a simple index of abundance and can be extrapolated to longer transects (2). |
| - Mark capture-recapture: Animals are physically captured, marked (e.g. with tags or dyes), released, and recaptured (physically or seen from the distance). The number of marked individuals together with the ratio of marked to unmarked individuals during the recapturing is used to estimate population size (3,4). |
| - Photographic mark-capture recapture: Animals are not physically captured and marked, but recorded and photographed, and re-identified from photographs (e.g. shape, color, and using natural markings) during the “recapturing”. The number of first photographed individuals together with the ratio of re-identified is used to estimate population size (5). |
| - Distance sampling technique: Observers walk along previously defined transect lines, recording the distance from the line to any animals they see. Applying a detection function fitted to the recorded distances of animals seen from the line, the number of animals missed during the survey is estimated, leading to an estimation of the density and abundance of the population in the surveyed area (1,6). |
| - Dog:human ratio: Data on the number of humans and the number of dogs is collected, e.g. during household surveys, and the ratio of dogs to humans is calculated. Human population census data are received from other sources (e.g. national databases, surveys), and the dog:human ratio is applied to estimate the dog population size of the entire area (7). |
| - Spatial models: Dog survey data detailing dog population characteristics, such as ownership trends and movement limitations are utilized to map the spatial distribution of dog populations and forecast the number of dogs in each region and nationwide (8). |
| **Dog movement techniques** |
| - Minimum Convex Polygon (MCP): The outermost locations of the geographical data points collected by GPS units or observation form a convex polygon, with the area inside the polygon representing the home range (9). One of the earliest and simplest methods to estimate an animal's home range. |
| - Kernel technique: A kernel is placed over each geographical data point collected by GPS units or observation and summing them up, produces a smooth density surface (Worton, 1989). The home range is calculated as the area within a certain isopleth of the overall kernel. |
| - Biased Random Bridges (BRB): The BRB is a kernel-based method that build kernel over movement tracks (a line between two consecutive geographical data point) rather than over single geographical data point (11). More specifically, it uses random movement bridges between consecutive locations, with a bias towards remaining in areas of previous high utilization. The method also accounts for temporal autocorrelation. |
| - Time Local Convex Hull (T-LoCoH): T-LoCoH improves on the LoCoH method, which identifies each point's nearest neighbors and then creates density-sorted convex hulls. By integrating time with space using a scaling that correlates distance and time to the individual's characteristic velocity, T-LoCoH generates hulls that are localized in both spatial and temporal dimensions. These hulls are progressively merged, and when they cover a specified percentage of points, the union is saved as an isopleth (12). |
| - Mixed effects logistic regression for spatial data: This method uses a statistical mixed effect logistic regression model, with the random effect representing a spatial component of the dataset, to account or spatial autocorrelation (13). Different model types are available that can also account for within-individual spatial auto-correlation and dependency between individuals (14). |
| - Social network analysis (SNA): SNA involves a set of techniques (not further detailed here) to study the interactions, i.e. contacts, between individuals, or on the general connectedness of the population (15). |

*
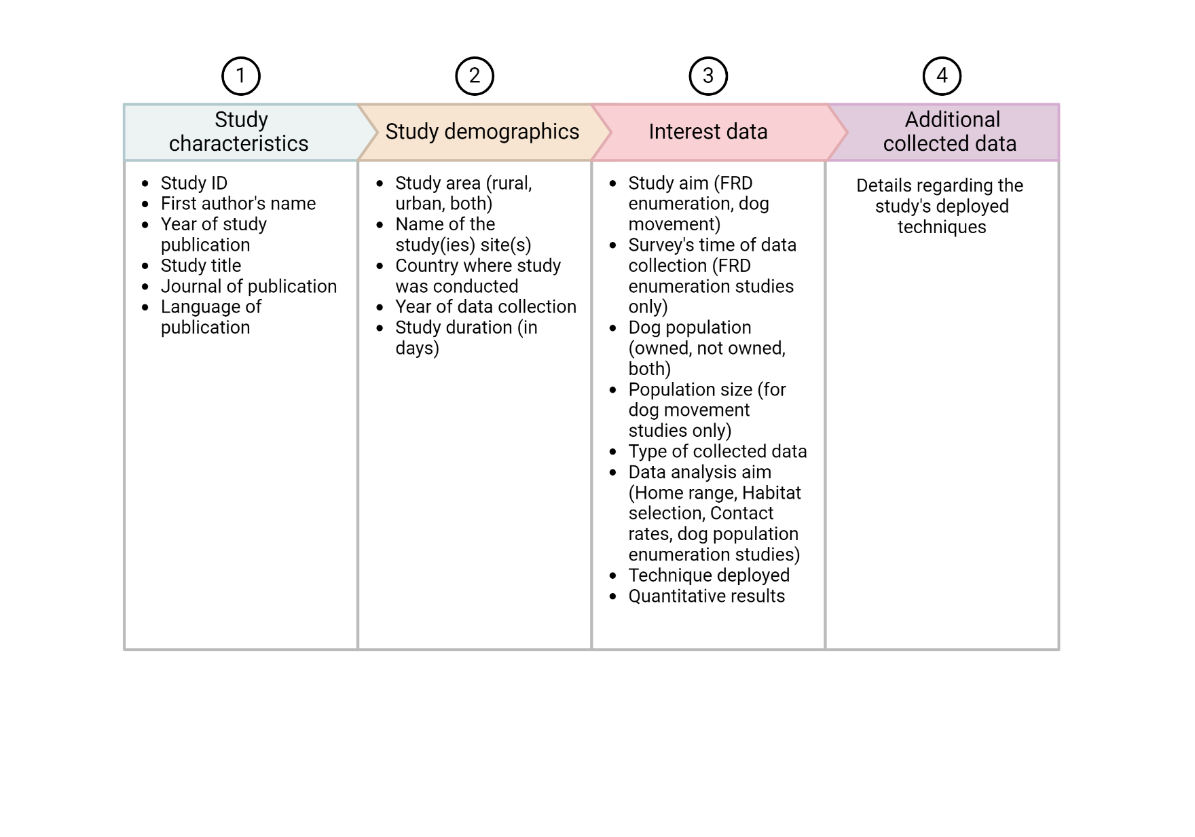
*

Figure S1 - Data extraction variables for the scoping review on free-roaming dogs.

Table S5 – Brief description of the main funding sources deployed in the included articles.

| Funding Source | Article ID |
| --- | --- |
| Government Grants | 8,19,21,51, 54,58,16,12,33,29,27,24,38,45,36,50,47,18 |
| University or Institutional Funding | 3,5,19,52,55,56,59,17,11,22,32,27,23,50,48,18 |
| Nonprofit and Charitable Organizations | 1,2,7,5,10,17, 15,12, 52,59,31,32,23,24,40,39,49,48 |
| Unfunded Research | 4,20,53,57,28,35,37,44,42,43,46 |
| International Organizations | 6,21,13,46,38,50 |

References:

1. Buckland ST, Anderson DR, Burnham KP, Laake JL. Distance Sampling: Estimating Abundance of Biological Populations. Biometrics. London: Chapman and Hall; 1993. 0–446 p.

2. Spurr EB, Borkin KM, Drew KW. Line-transect distance sampling compared with fixed-width strip-transect counts for assessing tomtit (Petroica macrocephala) population trends. N Z J Ecol [Internet]. 2012 [cited 2024 Jul 1];36(3). Available from: http://www.newzealandecology.org/nzje/

3. Lincoln FC. CALCULATING WATERFOWL ABUNDANCE ON THEBASIS OF BANDING RETURNS. UNITED STATES DEPARTMENT OF AGRICULTURE. 1930;(118).

4. Petersen CGJ. The yearly immigration of young plaice in the Limfjord from the German sea. Rept Danish Biol Sta [Internet]. 1896 [cited 2024 Jun 3];6(1):1–48. Available from: https://cir.nii.ac.jp/crid/1571698599852664192

5. Karanth KU. Estimating tiger Panthera tigris populations from camera-trap data using capture—recapture models. Biol Conserv. 1995 Jan 1;71(3):333–8.

6. Thomas L, Buckland ST, Burnham KP, Anderson DR, Laake JL, Borchers DL, et al. Distance sampling. In: El-Shaarawi AH, Piegorsch WW, editors. Encyclopedia of Environmetrics. 2002. p. 544–52.

7. World Health Organization/World Society for the Protection of Animals. Guidelines for dog population management. 1990.

8. Thanapongtharm W, Kasemsuwan S, Wongphruksasoong V, Boonyo K, Pinyopummintr T, Wiratsudakul A, et al. Spatial Distribution and Population Estimation of Dogs in Thailand: Implications for Rabies Prevention and Control. Front Vet Sci [Internet]. 2021 Dec 21 [cited 2024 Jul 17];8:790701. Available from: www.frontiersin.org

9. Mohr CO. Table of Equivalent Populations of North American Small Mammals. American Midland Naturalist. 1947 Jan;37(1):223.

10. Worton BJ. Kernel methods for estimating the utilization distribution in home- range studies. Ecology. 1989;70(1):164–8.

11. Benhamou S. Dynamic Approach to Space and Habitat Use Based on Biased Random Bridges. PLoS One [Internet]. 2011 [cited 2024 Jun 3];6(1):e14592. Available from: https://journals.plos.org/plosone/article?id=10.1371/journal.pone.0014592

12. Lyons AJ, Turner WC, Getz WM. Home range plus: A space-time characterization of movement over real landscapes. Mov Ecol [Internet]. 2013 Jul 3 [cited 2024 Aug 5];1(1):1–14. Available from: https://movementecologyjournal.biomedcentral.com/articles/10.1186/2051-3933-1-2

13. Duchesne T, Fortin D, Courbin N. Mixed conditional logistic regression for habitat selection studies. Journal of Animal Ecology. 2010 May;79(3):548–55.

14. Kanankege KST, Alvarez J, Zhang L, Perez AM. An Introductory Framework for Choosing Spatiotemporal Analytical Tools in Population-Level Eco-Epidemiological Research. Front Vet Sci [Internet]. 2020 Jul 7 [cited 2024 Jul 16];7:532589. Available from: www.frontiersin.org

15. Wasserman S, Faust K. Social Network Analysis: Methods and Applications [Internet]. Cambridge University Press; 1994 [cited 2024 Jun 3]. Available from: https://www.cambridge.org/core/books/social-network-analysis/90030086891EB3491D096034684EFFB8
